# Two Insertion/Deletion Variants within *SPAG17* Gene Are Associated with Goat Body Measurement Traits

**DOI:** 10.3390/ani9060379

**Published:** 2019-06-21

**Authors:** Sihuan Zhang, Enhui Jiang, Ke Wang, Yu Zhang, Hailong Yan, Lei Qu, Hong Chen, Xianyong Lan, Chuanying Pan

**Affiliations:** 1 College of Animal Science and Technology, Northwest A&F University, Yangling 712100, China; sihuanzhang1990@163.com (S.Z.); jiangenhui110@gmail.com (E.J.); lp_wangke@163.com (K.W.); 18829354069@163.com (Y.Z.); ylhailong@126.com (H.Y.); chenhong1212@263.net (H.C.); 2 Shaanxi Provincial Engineering and Technology Research Center of Cashmere Goats, Yulin University, Yulin 719000, China; ylqulei@126.com; 3 Life Science Research Center, Yulin University, Yulin 719000, China; 4 School of Life Sciences, Sun Yat-sen University, Guangzhou 510275, China

**Keywords:** goat, *SPAG17*, insertion-deletion (indel), body measurement traits, body height

## Abstract

**Simple Summary:**

Sperm-associated antigen 17 (*SPAG17*) is a reproduction and skeletal development related gene. This study aimed to identify crucial insertion-deletion (indel) variations, which influence the body measurement traits of goats. Two intronic indels (14 bp and 17 bp indels) were identified by sequencing. In Shaanbei white cashmere goat (SBWC), the different genotypes of the 14 bp indel were markedly associated with goat body height, chest width, body length, and chest depth. The genotypes of the 17 bp indel were significantly associated with body height and chest width. The different combined genotypes were significantly associated with body height and chest width of SBWC and ten traits of Hainan black goat. These results suggested that the 14 and 17 bp indels within *SPAG17* can be used in goat growth related traits marker-assisted selection breeding, especially body height.

**Abstract:**

Sperm-associated antigen 17 (*SPAG17*) gene encodes a multifunctional cytoplasmic protein, which influences not only reproduction but also skeletal development related body measurement traits, especially body height. Thus, this study aimed to identify crucial insertion-deletion (indel) variations, which influence the body measurement traits of goats in large goat populations (n = 1725). As a result, two intronic indels (14 bp and 17 bp indel) were identified by sequencing. For the two indel loci, the distributions of genotypes and alleles were significantly different between the Shaanbei white cashmere goat (SBWC) and the Hainan black goat (HNBG). In SBWC goats, the different genotypes of the 14 bp indel were markedly associated with goat body height, chest width, body length and chest depth. The genotypes of the 17 bp indel were significantly related to body height and chest width. At the two loci, for all seven analyzed traits of SBWC goat, the growth data of DD homozygotes were the worst, which means that the 14 bp insertion and the 17 bp deletion were beneficial and detrimental variations, respectively. Moreover, the combined genotypes were significantly related to body height and chest width of SBWC goats and ten traits of HNBG. These results suggested that the 14 and 17 bp indels within *SPAG17* can be used in goat growth related traits marker-assisted selection breeding, especially body height.

## 1. Introduction

Growth and development of goats are important factors that influence the developing of the goat industry. The body measurement traits data of goats directly reflect the body size, structure and development, which are closely related to the physiological function and production performance of goats. In the goat industry, the body measurement traits data could be used to guide the scientific raising and breeding of goats. Marker-assisted selection (MAS) is a fast, dependable and feasible breeding method, which is based on the significant variation loci in crucial genes. Thus, identifying crucial genes and markers associated with goat economic traits, such as growth and development, would lay the foundation for goat MAS breeding and assist in the genetic selection of goats.

Sperm-associated antigen 17 (*SPAG17*) plays vital roles in the function and structure of motile cilia [1,2]. It has been shown to play a variety of biological functions in reproduction. *Spag17* knockout mice are infertile due to a severe defect in germ cell differentiation [3]. A homozygous mutation (R1448Q) in *SPAG17* gene was identified in an asthenozoospermia patient, which suggested *SPAG17* may be a new pathogenic gene causing asthenozoospermia [4]. The transcript level of bovine *SPAG17* in pretransfer endometrial and embryo is related to pregnancy success and calf delivery [5]. A novel testis-specific splice variant of *SPAG17* is potentially applicable as immunotherapeutic targets and serologic biomarkers for cancer-testis [6]. 

Additionally, *SPAG17* plays a number of roles outside reproduction. *SPAG17* is relevant to primary ciliary dyskinesia, which is characterized by disrupted cilia motility in the lungs, trachea and brain [7,8]. Further, growing research has found that *SPAG17* regulates skeletal growth and mineralization [9,10]. The primary cilia in chondrocytes, osteoblasts and embryonic fibroblasts of *SPAG17* knockout mice are shorter and fewer than in wild-type mice, and *SPAG17* mutation shortens the length of hind limbs in mutant mice [7]. In 8182 of the European American children population tested, a height-associated locus of rs17038164 in *SPAG17* was identified [11]. In 8842 of the adult Korean population tested, one locus rs17038182 in *SPAG17* was found to be associated with idiopathic short stature [12]. The rs12735613 in *SPAG17* was identified to influence adult height [13]. Furthermore, rs9428104 SNP in *SPAG17* was related to height in individuals of European descent [14]. *SPAG17* rs7536458 SNP was associated to infant length [15]. Thus, *SPAG17* may play a crucial role in body measurement traits, especially body height.

Considering the functions of *SPAG17* on development, this study aimed to reveal the significant genetic variations in the goat *SPAG17* gene and investigate their effects on the body measurement traits of two Chinese goat breeds, Shaanbei white cashmere goat (SBWC) and Hainan black goat (HNBG). SBWC is a crossbreeding cashmere and meat dual-purpose breed, using Liaoning cashmere goats as the male parent and Shaanbei black goats as the female parent. Based on the measurement in 2016, the average body weight of SBWC male and female goats are 41.6 kg and 28.6 kg, respectively; the clear cashmere rate is up to 61.58%; and the average lambing rate of ewes is approximately 108% [16]. Some new breeding technologies have been used to further improve the production level of SBWC, such as gene editing [17]. HNBG, a valuable variety resource for large-scale breeding in tropical areas of China, is the only local goat breed in the Hainan province. It has a long history for raising HNBG, which can trace back to more than 1700 years ago. HNBG is a famous meat type of goat, which products tastes good and fat is evenly distributed throughout the meat. The reproductive performance of HNBG is strong, and the ewe lambing rate is up to 155% [18]. At present, the hybridization and improvement of HNBG are using Boer goats, Nubian black goats and Saanen dairy goats, and there is much room for the improvement of HNBG. The results of this study will provide molecular genetic markers for breeding high quality goats to benefit the goat industry.

## 2. Materials and Methods 

### 2.1. Ethics Statement

Experimental animals and procedures performed in this study were approved by the Faculty Animal Policy and Welfare Committee of Northwest A and F University under contract (NWAFU-314020038). The care and use of experimental animals fully complied with local animal welfare laws, guidelines and policies.

### 2.2. Sample Preparation and Data Collection

A total of 1725 ear samples were collected from random samples of Chinese indigenous well-known goat breeds: Shaanbei white cashmere goat (n = 1510) and Hainan black goat (n = 215). The meat and cashmere dual-purpose SBWC goats are all healthy adult females (2–3 years old) from a big population and reared in Shaanbei white cashmere goat breeding farm in Yulin city, Shaanxi Province. The ear tissue samples were collected after body size measurements in July 2016, 2017, and 2018. The growth related trait values were measured by technicians in the breeding farm, including hip height, body height, body length, chest width, chest depth, chest circumference and circumference of the cannon bone. According to the farm records, the measured goats were unrelated [19,20,21]. The meat-use breed HNBG adult female (2–3 years old) samples were collected from a native breeding farm in Zanzhou country of Hainan province, P.R. China. All the HNBG are healthy and unrelated. The data of HNBG includes eight body measurement traits, which were recorded in February 2009 [22]. The body measurement trait index were calculated based on the measured body traits, and the calculation methods were as follows: Body trunk index = heart girth / body length × 100, body length index = body length / body height × 100, heart girth index = heart girth / body height × 100; cannon circumference index = cannon circumference / body height × 100, chest width index = chest width / chest depth × 100, thurl width index = chest width/thurl width × 100 [23]. All DNA was extracted using the high salt-extraction method [24].

### 2.3. Primers Designing and Genotyping

According to the goat *SPAG17* sequence in NCBI (Accession number: NC_030810), six pairs of primers were designed to identify novel insertion-deletions (Indels) (Table 1). As this study aimed to detect the indels using an easy-to-operate, rapid, inexpensive, and exact PCR and agarose gel electrophoresis methods, only indels larger than 6 bp were listed as candidates. A 12.5 μL PCR reaction was performed, consisting of 10 ng genomic DNA, 0.5 μL of each primer, 6.25 μL 2×Taq Master mix (BioLinker, Shanghai, China) and ddH_2_O (added ddH_2_O up to 12.5 μL). The touch-down PCR program (68–55 °C) was performed [25]. The indel variations were identified by sequencing (Sangon Biotech, Shanghai, China) and all available individuals were genotyped using 3.5% agarose gel electrophoresis. 

### 2.4. Statistical Analysis of Results

The Hardy-Weinberg equilibrium (*HWE*) was analyzed by the SHEsis program [26]. The population genetic parameters, homozygosity (*Ho*), effective allele numbers (*Ne*) and polymorphism information content (*PIC*) were computed using Nei’s methods [27]. To analyze the genotypic and allelic frequency distributions of indels in different breeds, a chi-square test was performed. All the goats used in this study were unrelated, 2–3 years old, healthy, non-pregnant female, and the different breeds were raised in their respective farms and analyzed separately. The statistical analyses indicated that the age (2 and 3 years old) of goats had no clear influence on the various traits in the two analyzed populations. Therefore, this study used the reduced linear model to determine the relationship between genotypes and the various body measurement traits. The basic linear model was as follows:

Y_i_ = u + G_i_ + e

where Y_i_ was the trait measured data for each animal; u was the over mean for each trait; G_i_ was the effect of genotype and e was the random error. The association analysis was performed with SPSS 19.0 software by One-Way ANOVA followed by Post Hoc Multiple Comparisons [28].

### 2.5. The Linkage Disequilibrium and Combined Genotypes Analysis

The linkage disequilibrium analysis on the two indels was performed using the SHEsis online platform (http://analysis.biox.cn). The case of D’ (0 < D’ < 1) and r^2^ (0 < r^2^ < 1) indicate the linkage degree between the two loci. The D’ = 1 and r^2^ = 1 suggest the loci are in perfect linkage, and r^2^ > 0.33 indicates sufficiently strong linkage disequilibrium. When D’ < 1, it is hard to make a judgment whether the loci are linked according to the D’ value, as the practical meaning of the D’ value is easily exaggerated when the sample size is not big enough or the frequency of one allele is low [26,28,29]. 

## 3. Results

### 3.1. Identification of Indel Variations and Genotyping

**Figure 1:**
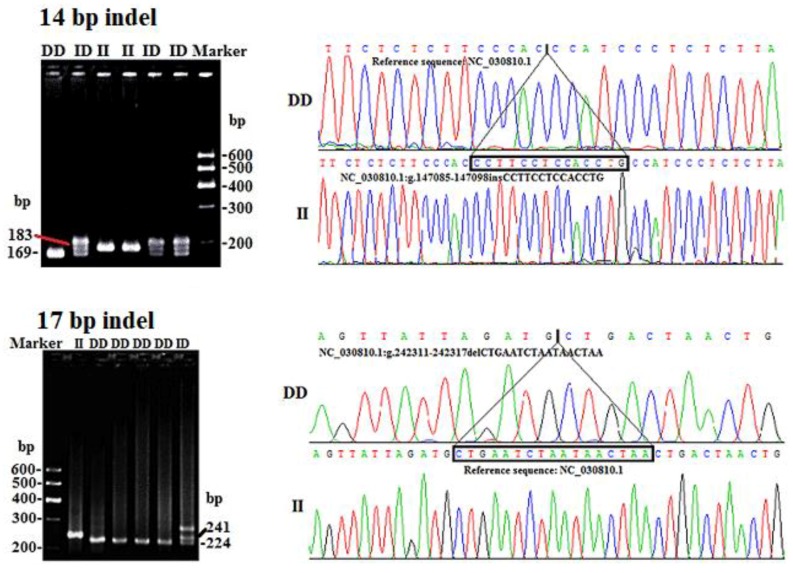
The sequencing map and electrophoresis pattern of 14 bp and 17 bp indel variants in goat *SPAG17* gene. II, homozygote insertion genotype; DD, homozygote deletion genotype; ID, heterozygote genotype. The sequence with the black border is the difference sequence fragment. The top band in the electrophoresis pattern of ID represented heteroduplex. Marker: 600-500-400-300-200-100 bp.

Combining DNA re-sequencing results of several goat breeds [30] and NCBI-dbSNP database, two indel loci, NC_030810.1:g.147085-147098insCCTTCCTCCACCTG (14 bp indel) and NC_030810.1:g.242311-242317delCTGAATCTAATAACTAA (17 bp indel) in goat *SPAG17* were identified using P4 and P6 primer pairs (Figure 1). The accession numbers of the 14 bp indel and 17 bp indel in NCBI-dbSNP database are rs659761737 in intron 22 and rs647063466 in intron 47 [Capra hircus], respectively. The 14 bp indel generated three genotypes: Homozygote insertion (II, 183 bp), homozygote deletion (DD, 169 bp) and heterozygote (ID, heteroduplex, 183 bp and 169 bp). The 17 bp indel also generated three genotypes: II (241 bp), DD (224 bp) and ID (heteroduplex, 241 bp and 224 bp (Figure 1).

### 3.2. Genetic Parameters Analysis

According to the genotyping results in goats, the genotypic distribution, allelic frequencies and population genetic parameters were calculated (Table 2). For the 14 bp indel, the frequency of the D allele was higher than the I in the two goat populations, and the frequency of DD was higher than that of II and ID genotypes. The frequency of DD in SBWC goats was up to 0.847. However, in HNBG, the three genotypes distributed evenly, and the frequencies of DD, ID and II were 0.398, 0.384, and 0.218, respectively. For the 17 bp indel, the frequency of DD was also the highest in the SBWC goat population. However, in the HNBG population, the frequency of ID was the highest (0.545). Furthermore, it was observed that the 14 bp indel belonging to low genetic diversity (*PIC* < 0.25) in SBWC goats, and medium genetic diversity (0.25 ≤ *PIC* ≤ 0.5) in the HNBG population. The 17 bp indel belongs to the medium genetic diversity in the two detected populations (Table 2). Interestingly, a chi-square test found that at the two loci, the distributions of genotypes and alleles were significantly different between the two types of breeds (*p* < 0.001), implying that they might be quantitative trait nucleotides (QTNs) with specific effects on producing cashmere or meat (Table 2). 

### 3.3. Association Analysis of Genotypes and Body Measurement Traits

**Figure 2:**
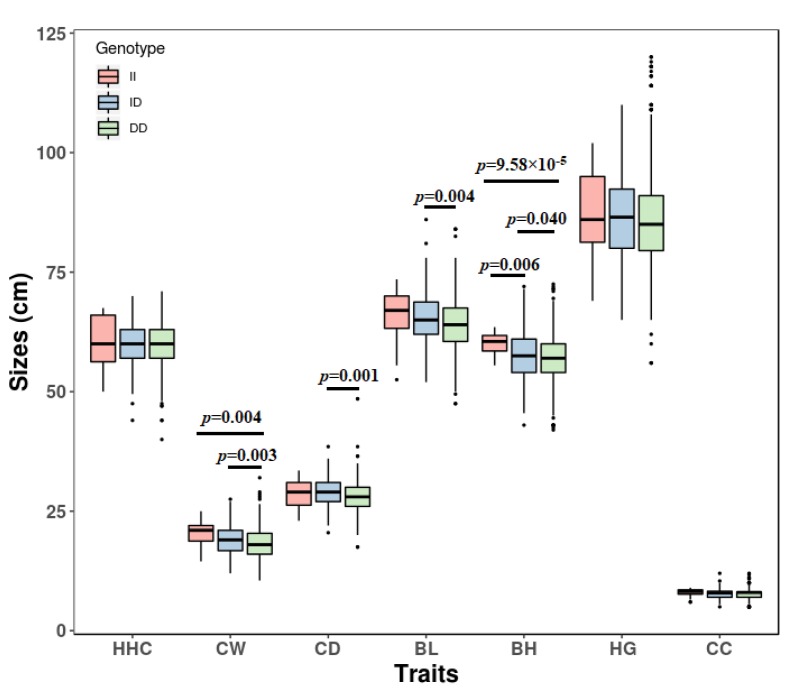
The association of goat *SPAG17* 14 bp indel and body measurement traits of Shaanbei cashmere goats. The *P* values were the results of post hoc multiple comparisons between genotypes. II, homozygote insertion genotype; DD, homozygote deletion genotype; ID, heterozygote genotype. HHC, height at hip cross; CW, chest width; CD, chest depth; BL, body length; BH, body height; HG, heart girth; CC, cannon circumference.

**Figure 3:**
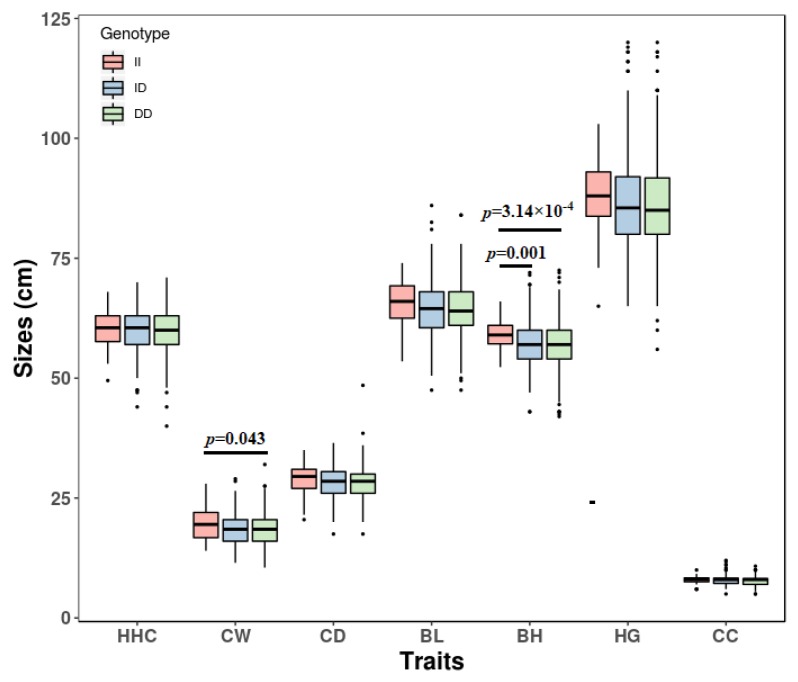
The association of goat *SPAG17* 17 bp indel and body measurement traits of Shaanbei cashmere goats. The *p* values were the results of post hoc multiple comparisons between genotypes. II, homozygote insertion genotype; DD, homozygote deletion genotype; ID, heterozygote genotype. HHC, height at hip cross; CW, chest width; CD, chest depth; BL, body length; BH, body height; HG, heart girth; CC, cannon circumference.

In SBWC goats, the association analysis between body measurement traits and genotypes demonstrated that different genotypes of 14 bp indel were markedly associated with body height (*p* = 4.12 × 10^−4^), chest width (*p* = 3.05 × 10^−4^), chest depth (*p* = 0.006) and body length (*p* = 0.003) (Table 3, Figure 2). Interestingly, for all analyzed traits (body height, chest width, body length, chest depth, heart girth, cannon circumference, and height at hip cross), the growth data of DD homozygotes of the 14 bp indel were the worst, and ID and II homozygotes were better. These results mean that the 14 bp insert mutation was a beneficial variation. At the 17 bp indel locus, the genotypes were significantly related to body height (*p* = 0.006) and chest width (*p* = 0.043) of SBWC goats (Table 3, Figure 3). For all analyzed traits, II homozygotes had the best growth data, but DD homozygotes had the worst growth data at the 17 bp indel locus. These results demonstrated that the 17 bp deletion mutation was a detrimental variation. However, the association analysis found that the genotypes were not associated significantly with growths traits of HNBG at the both indel loci (*p* > 0.05, Table 4). 

### 3.4. The Linkage Disequilibrium and Combined Genotypes Analysis

The linkage disequilibrium analysis results of the two indel loci in SBWC and HNBG breeds were presented by D’ and r^2^. In SBWC goats, the D’ value is 0.086, and r^2^ value is 0.003, and in HNBG, the D’ value is 0.017, and r^2^ value is close to 0. These results suggested that the goat *SPAG17* 14 bp and 17 bp indels are not linked in SBWC goats and HNBG breeds. Considering the sample size of SBWC goats was large (n = 1510), and the genotypes were distributed evenly in HNBG, the authors analyzed the relationship between combined genotypes of the 14 bp and 17 bp indels and body measurement traits of SBWC goats and HNBG. The combined genotypes with low frequencies (frequency < 0.05) were eliminated in the association analysis. As a result, in SBWC goats, four combined genotypes were reserved for association analysis, and different combined genotypes were significantly related to body height (*p* = 0.009) and chest width (*p* = 0.011) (Table 5). In HNBG, nine combined genotypes were significantly associated with ten body measurement traits (Table 6). These results further proved the crucial roles of *SPAG17* on body measurement traits, especially body height of SBWC goats.

## 4. Discussion

Genetic variation refers to the heritable variation which occurrs on genomic DNA molecules, containing the changes of base pair compositions or arrangements. This variation impels species to produce a variety of traits, which is essential for the continuation of the species and breeding. On a DNA level, genetic variation includes large DNA fragment variations (sizes range from Kb to Mb), that is, copy number variation (CNV), bases insertion or deletion (1 to 50 nucleotides), that is, insertion-deletion (indel) and single nucleotide polymorphism (SNP), etc. Among these variations, the indel is the easiest and the most cost-effective detected variation. When the mutation sequence is greater than 6 bases, it can be genotyped directly by PCR amplification and agarose gel electrophoresis. Thus, the crucial indel variation is more suitable for production practice, such as animal breeding. 

In this study, the authors uncovered two indels (14 bp indel and 17 bp indel) in *SPAG17* gene which significantly associated with goat body measurement traits, especially body height. This is consistent with previous research on other species, that *SPAG17* genetic variations play important roles in body height [11,12,13,14,15]. In SBWC, the 14 bp indel and the 17 bp indel loci were related to four and two body measurement traits, respectively. In HNBG, no significant association was identified. The distributions of genotypes and alleles were significantly different between the two type breeds. These results might be caused by the genetic background and the breeding of these two species. The SBWC is a new crossbreeding cashmere and meat dual-purpose breed, but the HNBG is a local meat-purpose goat breed in the Hainan province. 

The LD analysis results showed that the D’ and r^2^ values were very low (less than 0.33) in the two goat breeds, suggesting that the two loci are not linked in SBWC and HNBG breeds. Though the two loci are not linked, their mutation may have a superposition effect. Considering the sample size of SBWC goats was large (n = 1510), and the genotypes were distributed evenly in HNBG, the authors analyzed the relationship between combined genotypes and body measurement traits of these two breeds. The results showed the DD-DD combined genotype had the worst body measurement traits in SBWC, which was consistent with the association analysis results of genotypes (each locus) and body measurement traits. In the HNBG, no significant association was found in genotypes (each locus) and body measurement traits analyses, but in the combined genotype analyses, six body measurement traits and four body measurement trait indices were found significantly associated with combined genotypes. These results demonstrated that these two loci may have additive effects. Body measurement traits of goats have big influences on economic benefits in the animal industry. Identifying body measurement traits associated genes and genetic variations have important implications for goat marker-assisted selection breeding. These data suggested that the two indels might provide useful insights for goat growth related traits marker-assisted selection breeding, and lay the foundations for breeding of Shaanbei white cashmere goats. 

The indels that occur in the coding region of mRNA result in changes of protein, such as frameshift mutation, amino acid deficiency, etc. The indels are relatively common in non-coding regions rather than coding regions [31]. In this study, the two functional indels both occurred in the intron region. Many studies have found that variations in the intron have big influences on biological characters, and some are morbigenous by activating non-canonical splice sites or changing the splicing regulatory elements [32]. Van Laere et al. [33] uncovered that a nucleotide substitution in pig *IGF2* intron 3 caused a paternally expressed QTL affecting muscle and fat development by abrogating the interaction with a nuclear factor. In the dystrophin gene, a G to A transition at the fifth position of intron 32 (4518 + 5 G > A) inactivate a splice-donor site leading to transcript termination [34]. Wang et al. [35] found that a SNP (c. 1033 + 2184 C > T) in the intron 8 (the SNP located on the exonic splicing enhancer motif region) of dairy cow *CD46* gene cause alternative splicing. However, the mechanism of how the 14 bp indel and 17 bp indel in goat *SPAG17* gene influence the body measurement traits, especially body height, needs further study. The two indels in the goat *SPAG17* gene are associated with goat body measurement traits, so they can be used as molecular makers for Shaanbei white cashmere goat breeding.

## Figures and Tables

**Table 1:** Amplification PCR primer sequences of the goat *SPAG17* gene.

Primers	Primer Sequences (5’→3’)	Product Sizes (bp)	Location
P1	F: AAACGAAGCCTCCAAAGA	263	Intron 2
	R: TGATCCATCGACCTGTAAGA		
P2	F: TGAAGGTGAACTTCCGAATA	287	Intron 4
	R: ATGAAACCAGAGCCCAGA		
P3	F: TCAAGTTCAAGGTGGTTTCA	168	Intron 10
	R: CCTTCAGCCCATCACTTACT		
P4	F: GAGGGAATGTGAGCAGGAT	169	Intron 22
	R: TTTGATGACAAGGAAGGGA		
P5	F: GATTTGGCTGAGTTATACGAGG	184	Intron 42
	R: GTAGGCAAAATGCGAGGT		
P6	F: AAGTTCAGGGAGTGTTAAGGA	241	Intron 47
	R: CTGTGCCAGACAGATGGTC		

**Table 2:** Genotypic and allelic frequencies and population parameters of *SPAG17* 14 and 17 bp indels in goat breeds.

Loci/Breeds	Sizes (N)	Genotypic Frequencies			*p* Value ^e^	Allelic Frequencies		*p* Value ^f^	^a^ HWE *p* Values	Population Genetic Parameters		
		II	ID	DD		I	D			*Ho* ^b^	*Ne* ^c^	*PIC* ^d^
**14 bp indel**												
SBWC	1510	0.013	0.140	0.847	3.4 × 10 ^−70^	0.083	0.917	2.7 × 10 ^−83^	*p* < 0.05	0.848	1.179	0.140
HNBG	211	0.218	0.384	0.398		0.410	0.590		*p* < 0.05	0.519	1.926	0.365
**17 bp indel**												
SBWC	1191	0.036	0.354	0.610	1.4 × 10 ^−35^	0.213	0.787	1.9 × 10 ^−34^	*p* > 0.05	0.664	1.505	0.279
HNBG	211	0.223	0.545	0.232		0.495	0.505		*p* > 0.05	0.500	2.000	0.375

Note: SBWC, Shaanbei cashmere goat; HNBG, Hainan black goat; II, homozygote insertion genotype; DD, homozygote deletion genotype; ID, heterozygote genotype; ^a^ HWE, Hardy-Weinberg equilibrium; ^b^ Ho, observed homozygosity; ^c^ Ne, effective allele numbers; ^d^ PIC, polymorphism information content. The *p* value ^e^ and *p* value ^f^ represented the genotypic and allelic frequencies distribution different between the two breeds, respectively, which were calculated by Chi-Squared analysis.

**Table 3:** The association of *SPAG17* 14 and 17 bp indels and body measurement traits of Shaanbei cashmere goats.

Loci-Body Measurement Traits	Genotypes (Mean ± SE)			*p* Values
	II (n = 19)	ID (n = 211)	DD (n = 1278)	
14 bp indel				
Body height (cm)	60.11 ^A^ ± 0.57	57.89 ^B^ ± 0.34	56.99 ^B^ ± 0.12	4.12 × 10 ^−4^
Chest width (cm)	20.34 ^A^ ± 0.66	18.98 ^A^ ± 0.22	18.30 ^B^ ± 0.09	3.05 × 10 ^−4^
Chest depth (cm)	28.50 ^AB^ ± 0.74	28.75 ^A^ ± 0.20	27.99 ^B^ ± 0.10	0.003
Body length (cm)	65.84 ^AB^ ± 1.35	65.00 ^A^ ± 0.38	63.82 ^B^ ± 0.15	0.006
Heart girth (cm)	86.42 ± 2.13	86.42 ± 0.57	85.32 ± 0.27	0.270
Cannon circumference (cm)	7.91 ± 0.21	7.77 ± 0.06	7.70 ± 0.03	0.377
Height at hip cross (cm)	60.03 ± 1.18	60.04 ± 0.29	59.74 ± 0.13	0.643
**17 bp indel**	**II (n = 43)**	**ID (n = 421)**	**DD (n = 725)**	***p*** **Values**
Body height (cm)	59.13 ^A^ ± 0.46	57.15 ^B^ ± 0.22	57.07 ^B^ ± 0.17	0.006
Chest width (cm)	19.42 ^a^ ± 0.51	18.54 ^ab^ ± 0.15	18.42 ^b^ ± 0.12	0.043
Chest depth (cm)	28.69 ± 0.48	28.18 ± 0.17	28.17 ± 0.14	0.533
Body length (cm)	65.38 ± 0.78	64.25 ± 0.28	64.16 ± 0.20	0.363
Heart girth (cm)	87.36 ± 1.14	86.45 ± 0.45	85.68 ± 0.34	0.241
Cannon circumference (cm)	7.92 ± 0.12	7.85 ± 0.04	7.77 ± 0.03	0.215
Height at hip cross (cm)	60.61 ± 0.63	60.00 ± 0.22	59.66 ± 0.16	0.228

Note: Means with different superscripts within the same line represented differ significantly at *p* < 0.01 (A, B) or *p* < 0.05 (a, b) level. The *p* values are the results of One-Way ANOVA analysis. II, homozygote insertion genotype; DD, homozygote deletion genotype; ID, heterozygote genotype.

**Table 4:** The association of *SPAG17* 14 and 17 bp indels and body measurement traits of Hainan black goats.

Loci-Body Measurement Traits	Genotypes (Mean ± SE)			*p* Values
	II (n = 42)	ID (n = 76)	DD (n = 81)	
14 bp indel				
Body weight (kg)	29.71 ± 1.13	29.69 ± 0.68	29.44 ± 0.77	0.967
Body height (cm)	52.68 ± 0.54	53.93 ± 0.49	53.11 ± 0.41	0.201
Body length (cm)	56.42 ± 0.78	56.52 ± 0.49	56.49 ± 0.49	0.994
Heart girth (cm)	73.09 ± 1.00	73.56 ± 0.66	72.84 ± 0.69	0.757
Chest depth (cm)	26.76 ± 0.29	27.04 ± 0.24	26.71 ± 0.24	0.579
Chest width (cm)	14.96 ± 0.33	15.09 ± 0.17	15.29 ± 0.20	0.585
Hip width (cm)	13.75 ± 0.19	14.08 ± 0.16	13.72 ± 0.15	0.203
Cannon circumference (cm)	8.00 ± 0.11	7.93 ± 0.07	8.03 ± 0.07	0.610
Body trunk index	129.92 ± 1.55	130.39 ± 0.99	129.11 ± 0.88	0.646
Body length index	107.23 ± 1.27	105.08 ± 0.83	106.66 ± 0.97	0.304
Heart girth index	138.87 ± 1.56	136.67 ± 0.99	137.38 ± 1.19	0.510
Cannon circumference index	15.21 ± 0.21	14.74 ± 0.13	15.18 ± 0.15	0.050
Chest width index	55.85 ± 0.97	55.91 ± 0.53	57.34 ± 0.65	0.190
Thurl width index	108.80 ± 1.81	107.76 ± 1.23	111.80 ± 1.27	0.070
**17 bp indel**	**II (n = 44)**	**ID (n = 109)**	**DD (n = 46)**	***p*** **Values**
Body weight (kg)	30.09 ± 1.02	29.49 ± 0.57	29.35 ± 1.18	0.847
Body height (cm)	53.14 ± 0.53	53.38 ± 0.37	53.38 ± 0.64	0.937
Body length (cm)	56.86 ± 0.71	56.35 ± 0.40	56.43 ± 0.74	0.813
Heart girth (cm)	73.24 ± 0.96	73.45 ± 0.53	72.42 ± 1.03	0.632
Chest depth (cm)	26.80 ± 0.32	26.95 ± 0.19	26.65 ± 0.32	0.703
Chest width (cm)	15.28 ± 0.29	15.16 ± 0.15	14.98 ± 0.29	0.706
Hip width (cm)	13.79 ± 0.20	14.03 ± 0.12	13.54 ± 0.22	0.110
Cannon circumference (cm)	7.90 ± 0.11	8.05 ± 0.06	7.91 ± 0.10	0.293
Body trunk index	129.06 ± 1.36	130.60 ± 0.85	128.48 ± 1.15	0.314
Body length index	107.22 ± 1.33	105.88 ± 0.80	105.88 ± 1.02	0.626
Heart girth index	137.93 ± 1.47	137.92 ± 0.98	135.76 ± 1.29	0.426
Cannon circumference index	14.90 ± 0.19	15.14 ± 0.13	14.85 ± 0.15	0.320
Chest width index	57.04 ± 0.83	56.33 ± 0.49	56.30 ± 0.96	0.751
Thurl width index	111.12 ± 1.80	108.47 ± 1.05	110.95 ± 1.67	0.281

Note: The *p* values are the results of One-Way ANOVA analysis. II, homozygote insertion genotype; DD, homozygote deletion genotype; ID, heterozygote genotype.

**Table 5:** The association of goat *SPAG17* 14 and 17 bp indel combined genotypes and body measurement traits of Shaanbei cashmere goats.

Body Measurement Traits	Combined Genotypes (Mean ± SE)/Frequencies (Number)				*p* Values
	ID-ID 0.061 (71)	ID-DD 0.290 (339)	DD-ID 0.100 (117)	DD-DD 0.511 (598)	
Body height (cm)	58.02 ^a^ ± 0.63	56.93 ^a^ ± 0.24	58.01 ^b^ ± 0.43	56.83 ^b^ ± 0.18	0.009
Chest width (cm)	18.72 ^ab^ ± 0.39	18.46 ^ab^ ± 0.17	19.08 ^a^ ± 0.30	18.27 ^b^ ± 0.13	0.011
Chest depth (cm)	28.72 ± 0.35	28.14 ± 0.19	28.78 ± 0.26	28.18 ± 0.17	0.265
Body length (cm)	65.07 ± 0.74	64.02 ± 0.31	64.93 ± 0.48	63.95 ± 0.22	0.152
Heart girth (cm)	87.02 ± 0.93	86.27 ± 0.53	86.02 ± 0.80	85.65 ± 0.38	0.581
Cannon circumference (cm)	7.88 ± 0.12	7.83 ± 0.05	7.71 ± 0.07	7.79 ± 0.03	0.416
Height at hip cross (cm)	59.69 ± 0.53	60.05 ± 0.24	60.29 ± 0.35	59.53 ± 0.18	0.186

Note: Means with different superscripts within the same line represented differ significantly at *p* < 0.05 (a, b) level. The *p* values are the results of One-Way ANOVA analysis. DD, homozygote deletion genotype; ID, heterozygote genotype.

**Table 6:** The association of goat *SPAG17* 14 and 17 bp indel combined genotypes and body measurement traits of Hainan black goats.

Body Measurement Traits (n = 199)	Combined Genotypes (Mean ± SE)/Frequencies (Number)									*p* Value
	II-II 0.055 (11)	II-ID 0.096 (n = 19)	II-DD 0.060 (12)	ID-II 0.080 (16)	ID-ID 0.221 (44)	ID-DD 0.080 (16)	DD-II 0.086 (17)	DD-ID 0.231 (46)	DD-DD 0.091 (18)	
Body weight (kg)	30.23 ^abc^ ± 1.72	27.37 ^c^ ± 1.30	32.94 ^a^ ± 2.85	28.44 ^abc^ ± 1.33	30.95 ^ab^ ± 0.84	27.53 ^b^ ± 1.69	31.56 ^abc^ ± 2.03	29.00 ^abc^ ± 0.91	28.58 ^abc^ ± 1.70	0.022
Body height (cm)	52.18 ^ab^ ± 0.77	52.01 ^b^ ± 0.88	54.21 ^ab^ ± 1.01	52.36 ^b^ ± 0.79	54.60 ^a^ ± 0.62	53.64 ^ab^ ± 1.32	54.51 ^ab^ ± 1.00	52.79 ^b^ ± 0.50	52.61 ^ab^ ± 0.93	0.015
Body length (cm)	57.45 ^abcd^ ± 1.23	54.38 ^d^ ± 0.97	58.70 ^a^ ± 1.76	55.46 ^abcd^ ± 1.15	57.45 ^ab^ ± 0.59	55.00 ^bcd^ ± 1.12	57.80 ^abc^ ± 1.23	56.12 ^abcd^ ± 0.61	56.19 ^abcd^ ± 1.02	0.008
Heart girth (cm)	73.64 ^ab^ ± 1.93	71.69 ^ab^ ± 1.22	74.79 ^ab^ ± 2.34	72.16 ^ab^ ± 1.35	74.63 ^a^ ± 0.84	72.03 ^ab^ ± 1.63	74.01 ^ab^ ± 1.79	73.05 ^ab^ ± 0.82	71.19 ^b^ ± 1.54	0.045
Chest depth (cm)	26.53 ^ab^ ± 0.61	26.39 ^ab^ ± 0.37	27.56 ^a^ ± 0.62	26.31 ^ab^ ± 0.44	27.45 ^a^ ± 0.31	26.65 ^ab^ ± 0.58	27.44 ^a^ ± 0.59	26.70 ^ab^ ± 0.3	26.04 ^b^ ± 0.46	0.015
Chest width (cm)	15.31 ± 0.53	14.36 ± 0.38	15.58 ± 0.86	15.13 ± 0.40	15.30 ± 0.20	14.49 ± 0.35	15.41 ± 0.58	15.35 ± 0.25	15.01 ± 0.35	0.399
Hip width (cm)	13.56 ^ab^ ± 0.22	13.67 ^b^ ± 0.27	14.04 ^ab^ ± 0.47	13.78 ^ab^ ± 0.27	14.39 ^a^ ± 0.19	13.51 ^b^ ± 0.41	13.95 ^ab^ ± 0.44	13.83 ^b^ ± 0.18	13.23 ^b^ ± 0.27	0.002
Cannon circumference (cm)	8.14 ± 0.21	8.00 ± 0.16	7.88 ± 0.25	7.72 ± 0.18	8.06 ± 0.09	7.78 ± 0.14	7.92 ± 0.17	8.06 ± 0.08	8.06 ± 0.15	0.538
Body trunk index	128.29 ± 2.55	132.30 ± 2.57	127.64 ± 2.66	130.58 ± 2.70	130.10 ± 1.30	131.01 ± 1.67	128.12 ± 1.92	130.38 ± 1.18	126.79 ± 1.79	0.635
Body length index	110.19 ^a^ ± 2.22	104.88 ^ab^ ± 2.02	108.22 ^ab^ ± 2.21	106.01 ^ab^ ± 1.89	105.54 ^ab^ ± 1.13	102.86 ^b^ ± 1.60	106.44 ^ab^ ± 2.58	106.61 ^ab^ ± 1.32	107.00 ^ab^ ± 1.49	0.022
Heart girth index	141.05 ± 2.66	138.14 ± 2.18	138.02 ± 3.62	137.91 ± 2.05	136.99 ± 1.36	134.58 ± 1.96	135.94 ± 2.81	138.72 ± 1.72	135.31 ± 1.52	0.749
Cannon circumference index	15.61 ^ab^ ± 0.35	15.44 ^ab^ ± 0.34	14.50 ^c^ ± 0.31	14.74 ^abc^ ± 0.27	14.81 ^b^ ± 0.18	14.54 ^b^ ± 0.19	14.58 ^b^ ± 0.34	15.33 ^a^ ± 0.20	15.34 ^abc^ ± 0.24	0.028
Chest width index	57.68 ^ab^ ± 1.36	54.43 ^b^ ± 1.23	56.40 ^ab^ ± 2.51	57.48 ^ab^ ± 1.11	55.85 ^ab^ ± 0.67	54.54 ^ab^ ± 1.21	56.21 ^ab^ ± 1.68	57.58 ^a^ ± 0.79	57.80 ^ab^ ± 1.43	0.037
Thurl width index	113.03 ^ab^ ± 3.95	105.32 ^b^ ± 2.46	110.44 ^ab^ ± 3.24	109.91 ^ab^ ± 2.51	106.87 ^b^ ± 1.72	108.04 ^ab^ ± 2.42	111.01 ^ab^ ± 3.24	111.29 ^ab^ ± 1.51	113.88 ^a^ ± 2.96	0.022

Note: Means with different superscripts within the same line represented differ significantly at *p* < 0.05 (a, b) level. The *p* values are the results of One-Way ANOVA analysis. DD, homozygote deletion genotype; ID, heterozygote genotype.
